# Visible light electrochromism based on reversible dissolution/deposition of MnO_2_

**DOI:** 10.1515/nanoph-2023-0573

**Published:** 2023-10-30

**Authors:** Xuan Liu, Hanbing Wang, Junsen Zhong, Menghan Li, Rui Zhang, Dongjiang You, Lingyu Du, Yanfeng Gao, Litao Kang

**Affiliations:** College of Environment and Materials Engineering, Yantai University, Yantai 264005, China; College of Materials Science and Engineering, Shanghai University, Shanghai 200444, China; Anhui Polytechnic University, Wuhu, Anhui 241000, China

**Keywords:** electrochromic, MnO_2_, reversible electrodeposition, cycling stability

## Abstract

Electrochromic (EC) windows based on reversible metal electro-deposition/dissolution (RME) are exciting alternatives to static lighting control using blinds and shades. However, the difficulty of reversibly and uniformly electrodepositing large-area metal layers seriously hinders the development of RME-type EC windows. In this paper, we develop a novel EC system based on reversible oxide electro-deposition/dissolution (ROE) of MnO_2_, achieving a profound and bistable color change between rust brown and completely transparent. This ROE-MnO_2_ system exhibits a significantly high optical modulation amplitude (Δ*T*) without any deterioration within 1000 switching cycles (Δ*T*_550nm_ actually increases from 44.8 % up to 46.9 % after the cycling test), which is almost double compared to the traditional cation-insertion/extraction triggered MnO_2_ (RCI-MnO_2_) EC system. This work may inspire the exploration of novel ROE-type EC systems.

## 1 Introduction

In the past few decades, electrochromic (EC) materials have found many potential applications in fields such as smart windows, switchable mirrors, anti-glare eyewear, passive display, electronic tags, etc., thanks to their flexibly tunable optical properties, low operation voltages and small energy consumption [1–3]. Electrochemically, the EC abilities stem from the redox reaction of active materials [4–6]. The primary reaction mechanisms of EC materials include reversible metal electro-deposition/dissolution (RME, Cu [7], Ag [8, 9], Bi [10], etc., Table S1) or cation insertion/extraction (RCI) [11–14].

In RME-type EC devices, the metal cations in the electrolytes can be readily reduced and deposited on the surfaces of working electrodes (typically FTO or ITO transparent conductive glasses) as metallic layers by applying a proper negative potential, transforming the devices from clear to dark [15]. Applying a positive enough potential can electrochemically oxidize the metallic layers back into metal cations, and thereby bleach the devices [16]. Indeed, there are many metals that can be reversibly electro-deposited/dissolved within cheap and nontoxic aqueous electrolytes [17]. These metals always exhibit high extinction coefficients. Therefore, a uniform metal layer of only 20–30 nm thickness is enough to block out the sunlight, resulting in high optical contrast [18]. Since the metallic layers are *in-situ* deposited, the RME devices are always structurally simpler than their RCI counterparts, making the fabrication more efficient in both materials and preparation cost [17, 19].

The long-term operation of RME devices requires strict control over the metal nucleation/growth process, in order to ensure uniform deposition and avoid metal dendrite growth derived by “tip discharge effect” [20, 21]. For example, McGehee et al. decorated the surfaces of ITO glass substrate with Pt nanoparticles, which can effectively improve the deposition uniformity of the metal layers as heterogeneous nucleation seeds [21, 22]. Besides, electrolyte formulation [23], adoption of co-deposition system [7], and counter electrode design [20, 24] have also been explored to improve the cycling lifespans of the RME devices. Nevertheless, the dendrite growth driven by “tip discharge effect” is a self-reinforcing and chronic vicious circle, leaving the achievement of durable and large area metal deposition still a substantial challenge [2, 25], [26], [27].

RCI is another common EC mechanism, which works very like the ubiquitous lithium-ion batteries [28, 29]. When a proper electric field is applied, cations and electrons simultaneously insert into or extract from the EC materials, leading to profound color changes due to the optical effects of valence variation [2, 30, 31]. Take MnO_2_ as an example, its color switching (i.e., cation insertion/extraction) processes can be expressed as [32]:

(1) $$\text{Mn}{\text{O}}_{2}\left(\text{rust}\;\;\text{brown}\right)+x{\text{A}}^{+}+\text{x}{\text{e}}^{-}↔{\text{A}}_{x}\text{Mn}{\text{O}}_{2}\left(\text{pale}\;\;\text{yellow}\right)$$

where A^+^ represents the inserting cations, which are typically Li^+^ or K^+^. The commonly used electrolytes in the RCI-MnO_2_ systems are LiClO_4_-PC [33–35] and KCl–H_2_O solutions [32].

Owing to the obvious optical absorption of A_*x*_MnO_2_, the bleached state of this EC system is still tinted [34], which therefore seriously limits the optical modulation amplitude (Δ*T*). For this reason, MnO_2_ is usually combined with other EC materials (e.g., PANI [33], V_2_O_5_ [36], and PB [32, 35]) to synergistically achieve high performances.

In this paper, we develop a novel EC system based on reversible oxide electro-deposition/dissolution (ROE) reaction of MnO_2_. To activate this ROE reaction (Mn^2+^ + H_2_O – 2e^−^ ↔ MnO_2_ + 4H^+^) [37, 38], an acidic electrolyte containing 0.5 M H_2_SO_4_ is adopted to accelerate the reaction kinetics, achieving a profound color change from rust brown to completely transparent, along with more than doubled optical modulation amplitudes compared to the conventional RCI-MnO_2_ system (Δ*T*: 45 % vs. 22.5 % at 550 nm, or 88.14 % vs. 12.7 % at 420 nm). To further improve the cycling durability of this ROE system, 15 mM Fe^3+^ is introduced into the electrolyte as catalysis to facilitate the dissolution of MnO_2_, resulting in an impressive cycling stability without any EC performance deterioration within 1000 switching cycles (Δ*T*_550nm_ actually increases from 44.6 % up to 46.9 % after 1000 cycles). To demonstrate the practical application of this EC system, an ROE-MnO_2_ dynamic window is assembled with a Cu foil counter electrode, which achieves not only impressive EC performance but also decent energy storage ability. The establishment of this ROE-MnO_2_ EC system may inspire further exploration of novel EC systems.

## 2 Experimental

### 2.1 Establishment of ROE-MnO_2_ EC system

The ROE-MnO_2_ system was established in a two-electrode configuration, where a FTO glass (2.5 × 2.5 cm), a Cu foil and a 0.5 M H_2_SO_4_ + 1 M MnSO_4_ + 15 mM CuSO_4_ aqueous solution were employed as the working electrode, counter/reference electrode and electrolyte, respectively. CV tests indicate that Cu can be smoothly and reversibly striped/plated in this electrolyte (Figure S1), and therefore is used as the counter electrode to compensate charge. To demonstrate the practical application of this EC system, an ROE-MnO_2_ dynamic window was constructed with a FTO glass working electrode, a narrow Cu belt counter electrode. The Cu counter electrode was attached onto a piece of float glass. These two glasses were separated and adhered by 2-mm thick double-sided tapes (3 M Company), forming a double-paned EC device. Afterwards, the electrolyte was injected into the internal cavity of the device using a syringe.

### 2.2 Assembly of RCI-MnO_2_ dynamic window

**Preparation of MnO**_**2**_ **film**: The MnO_2_ film was pre-electrodeposited on a FTO glass at a constant potential of 0.6 V for 500 s by using an electrochemical workstation (CHI660E, Shanghai Chenhua Instruments, Inc.) [39]. The electrodeposition process was performed in a three-electrode configuration that contained a Pt foil (10 mm × 10 mm) counter electrode, an Ag/AgCl (3 M KCl) reference electrode and a FTO glass working electrode. In this electrodeposition experiment, a 0.1 M Mn(CH_3_COO)_2_ + 0.1 M Na_2_SO_4_ aqueous solution was used as the electrolyte. The as-prepared MnO_2_-coated FTO glass was rinsed with de-ionized water and dried at room temperature.

**Assembly of RCI-MnO**_**2**_ **dynamic window**: An RCI-MnO_2_ dynamic window was constructed with a MnO_2_-coated FTO glass working electrode and a narrow Cu belt counter electrode. In the cavities of this device, an organic electrolyte consisting of 1 M LiClO_4_ + 15 mM Cu(ClO_4_)_2_ in polycarbonate was injected with a syringe.

### 2.3 Material characterization

Surficial morphologies of the FTO glass substrate and the deposited MnO_2_ layers were observed by a JEOL JSM-7610F field emission scanning electron microscope (SEM). X-ray diffraction (XRD) patterns of the FTO glasses before and after MnO_2_ deposition were collected within 10–80° on a D/max-2500/PC X-ray diffractometer with Cu Kα radiation (*λ* = 0.1542 nm). XPS (X-ray photoelectron spectroscopy) spectra was collected on Thermo Scientific K-Alpha^TM+^ spectrometer respectively, in order to investigate the oxidation states of deposited layer.

### 2.4 Electrochemical and optical characterization

Transmittance tests were carried out on a Persee TU-1810 UV–Vis spectrophotometer within a wavelength range of 200∼1100 nm. All the transmittances are calibrated with a blank device as baseline. To test the EC performance and cycling durability, a CHI-660E electrochemical workstation were used to impose controlled stimulating voltages on the EC systems to activate the color switching reactions. Electrochemical tests of the ROE-MnO_2_ or RCI-MnO_2_ were performed in a two-electrode configuration, with a pure FTO glass and a Cu foil as the working and counter/reference electrode, respectively.

## 3 Results and discussion

**Figure 1:**
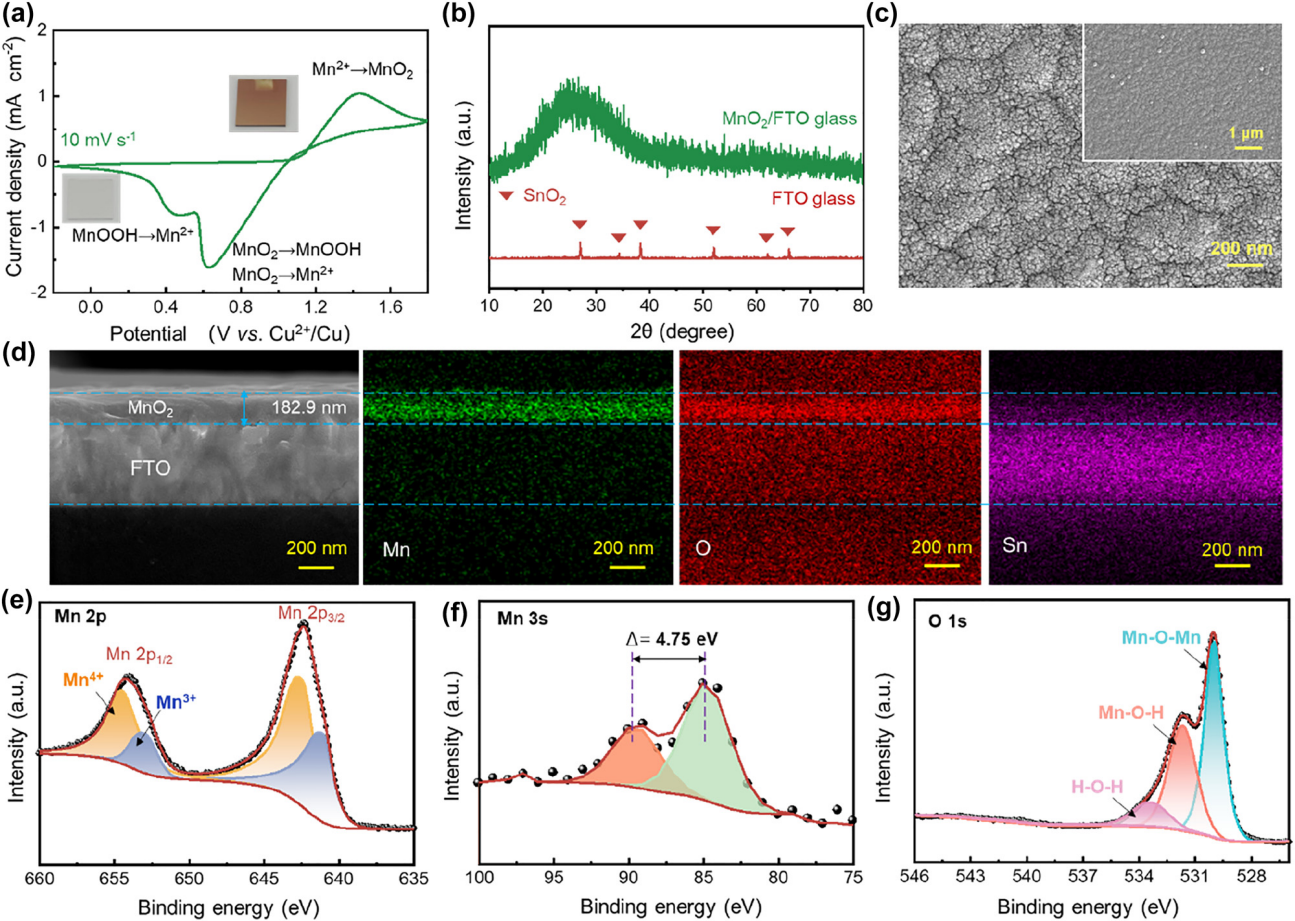
Characterizations of the ROE-MnO_2_ system: (a) photographs and typical CV curves swept at 10 mV s^−1^, (b) XRD pattern, (c) typical SEM images, (d) elemental mapping and (e–g) XPS spectra and fitting results of the *in-situ* deposited MnO_2_ in the colored state.

Figure 1a shows the cyclic voltammetry (CV) curve of the ROE-MnO_2_ system in a two-electrode configuration, which shows two cathodic peaks and an anodic peak at 0.4/0.6 V and 1.4 V, respectively. The anodic and cathodic processes can be expressed as [40]:

(2) $$\text{M}{\text{n}}^{2+}+{\text{H}}_{2}\text{O}↔\text{Mn}{\text{O}}_{2}+4{\text{H}}^{+}+2{\text{e}}^{-}$$

(3) $$\text{Mn}{\text{O}}_{2}+{\text{H}}^{+}+{\text{e}}^{-}↔\text{MnOOH}$$

(4) $$\text{MnOOH}+3{\text{H}}^{+}+{\text{e}}^{-}↔\text{M}{\text{n}}^{2+}+2{\text{H}}_{2}\text{O}$$

During the anodic process, the FTO working electrode is gradually tinted brown due to the deposition of amorphous MnO_2_ layer (Figure 1b–d) [41–44]. When the voltage sweeps back to low voltage, the layer and the brown tinct are completely wiped off by the cathodic process (insets of Figure 1a), indicating the occurrence of reverse reaction.

Figure 1c depicts the surficial morphology of the deposited layer, which is composed by uniform and tightly-packed nanoparticles. The successful deposition of MnO_2_ layer is also confirmed by energy dispersive spectroscopy (EDS). As shown in Figure 1d, the Mn and O elements uniformly distribute to form a 182.9 nm thick layer. Figure 1e presents the Mn 2p X-ray photoelectron spectrum (XPS) of the layer, which can be deconvoluted into two components: Mn^4+^ at 642.4 and 654.5 eV, and Mn^3+^ at 641.1 and 653.0 eV, respectively. The valence contributions of Mn^4+^ and Mn^3+^ are determined to be 63.65 % and 36.35 %, based on the areas of the deconvoluted peaks. The fitting result of the valence-sensitive Mn 3s peaks demonstrate a spin-energy splitting (ΔEs) of 4.75 eV (Figure 1f), corresponding to a Mn charge state of 3.57 [45], in good line with the Mn 2p-determined value. The deconvoluting result of the O 1s spectrum (Figure 1g) further indicates the co-existence of three Mn states: Mn–O–Mn peak from MnO_2_ (529.5 eV), Mn–OH peak from MnOOH (531.1 eV), and H–O–H peak from chemosorbed water (532.5 eV). According to the XPS results, the deposited layer should be nonstoichiometric manganese oxide (MnO_*x*_) that consists of amorphous MnO_2_, MnOOH and chemosorbed water. As expressed in Equations (3) and (4), MnOOH is an intermediate product in the electro-dissolution of MnO_2_ [40, 46].

**Figure 2:**
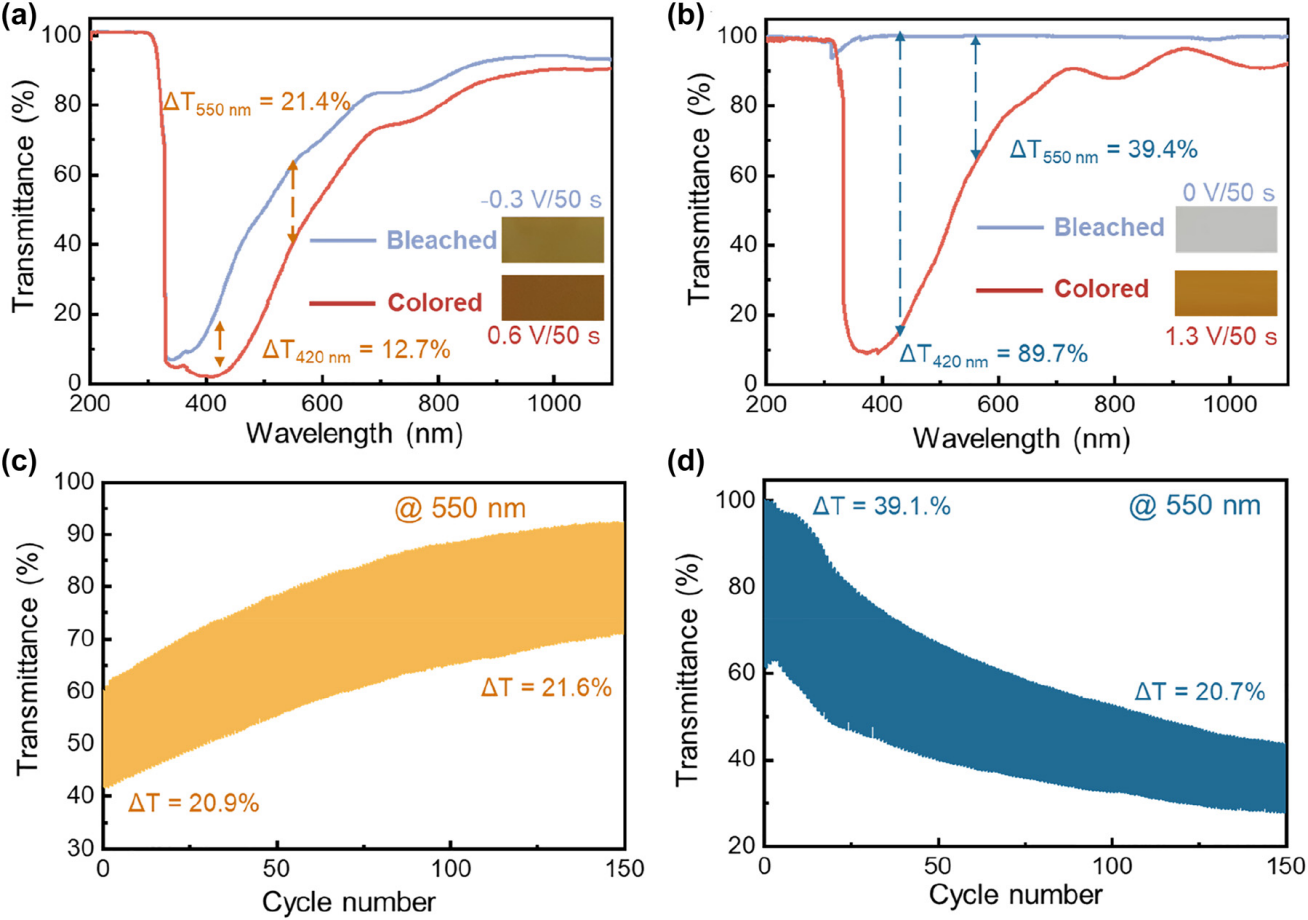
Electrochromic performance comparison of RCI- and ROE-MnO_2_ systems: (a and b) transmittance spectra and photographs (inset) in the colored and bleached states, (c and d) transmittance evolution curves at 550 nm during continuous color switching.

Figure 2a and b compare the transmittance spectra and photographs of the RCI- and ROE-MnO_2_ EC systems in their bleached and colored state, respectively. The RCI system delivers obviously smaller Δ*T* in the entire wavelength range, e.g., 12.7 % at 420 nm and 21.4 % at 550 nm, due to the relatively strong optical adsorption of its bleached state. In striking contrast, the ROE system exhibits much stronger color change between rust brow and transparent, thanks to the complete electro-deposition/dissolution of the MnO_2_ layer. As a result, the system achieves a significant Δ*T* of 89.7 % at 420 nm, or 39.4 % at 550 nm. However, both of these EC systems suffers from poor cyclic stability (Figure 2c and d). As shown in Figures 2c and S2, the transmittance of the RCI system undergoes a dramatic increase, even through its Δ*T*_550nm_ keeps relatively stable (increase from 20.9 % to 21.6 %), suggesting the occurrence of “ion trapping” process [47–49]. On the other hand, the ROE system’s Δ*T*_550nm_ gradually deteriorates from 39.1 % down to 20.7 % after 150 times continuous color switching. During cyclic color switching, the transmittances of the ROE system remarkably decrease regardless of the color state, due to the formation and accumulation of residual MnO_2_ deposits on the FTO glass substrate (Figure S3). During bleaching, the dissolution of MnO_2_/MnOOH may disconnect the deposits from the substrate, kinetically limiting the complete dissolution of the deposits due to electrically insulation [46].

**Figure 3:**
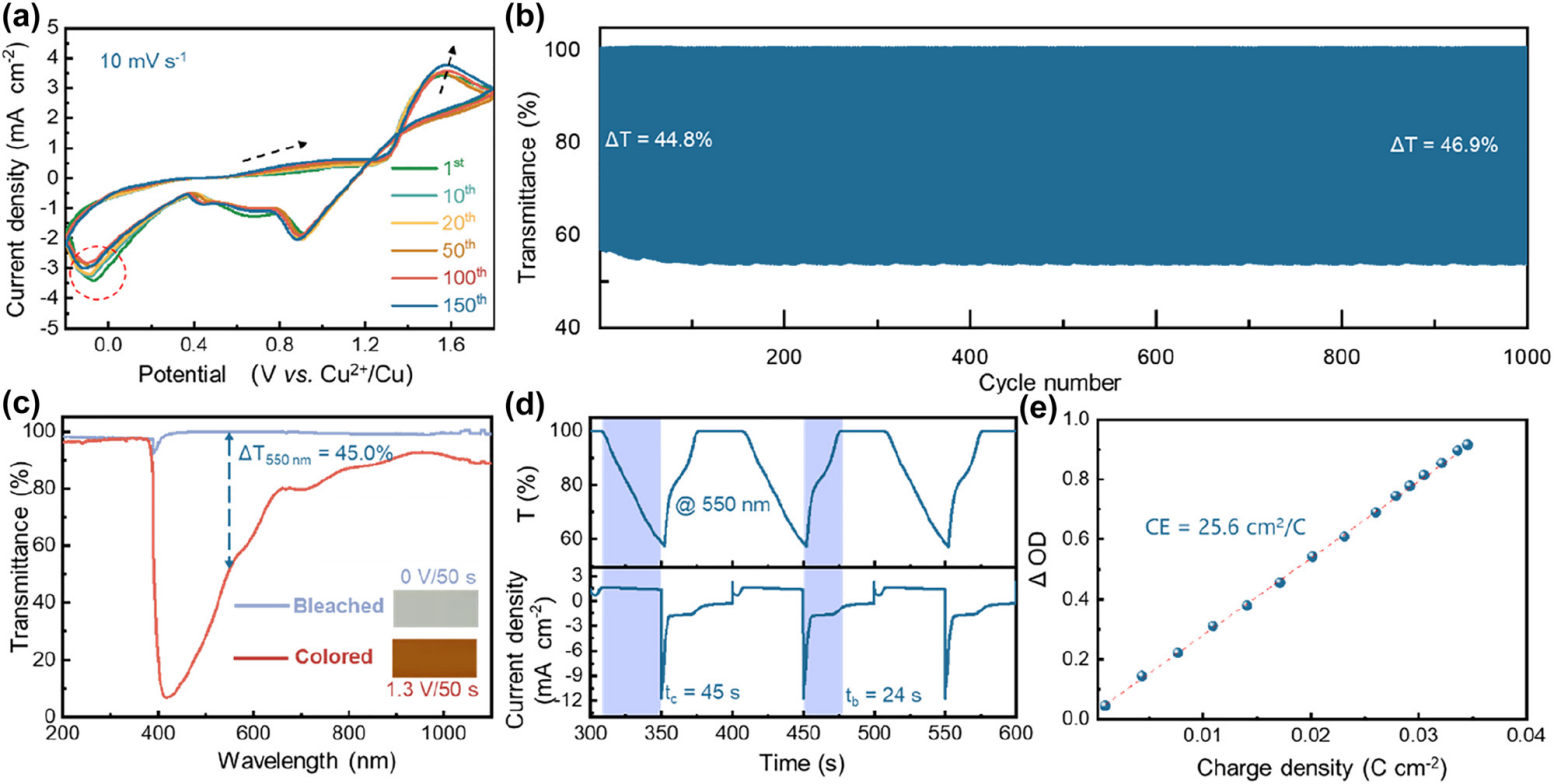
Electrochromic performances of the ROE-MnO_2_ system in the FeCl_3_-modified electrolyte: (a) typical CV curves swept at 10 mV s^−1^, (b) transmittance evolution curves at 550 nm during continuous color switching, (c) transmittance spectra and photographs (inset) in colored and bleached states, (d) transmittance evolution and current response curves showing the coloring/bleaching times, (e) optical density (ΔOD, @550 nm) versus charge density curves.

To facilitate the dissolution of MnO_2_, 15 mM FeCl_3_ is introduced into the electrolyte as a redox mediator. Fe^3+^/Fe^2+^ couple has a lower redox potential than the Mn^4+^/Mn^2+^ one (Figure 3a and S4) [46], therefore this couple can be used to accelerate the dissolution of MnO_2_ and MnOOH:

(5) $$\text{F}{\text{e}}^{3+}+2{\text{e}}^{-}\to \text{F}{\text{e}}^{2+}$$

(6) $$2\text{F}{\text{e}}^{2+}+\text{Mn}{\text{O}}_{2}+4{\text{H}}^{+}\to 2\text{F}{\text{e}}^{3+}+\text{M}{\text{n}}^{2+}+2{\text{H}}_{2}\text{O}$$

(7) $$\text{F}{\text{e}}^{2+}+\text{MnOOH}\to \text{F}{\text{e}}^{3+}+\text{M}{\text{n}}^{2+}$$

During the bleaching process, Fe^3+^ is firstly electrochemically reduced to Fe^2+^ (Equation (5)), which then reduces the residual MnOOH and MnO_2_ deposits into soluble Mn^2+^ (Equations (6) and (7), Figure S5), ensuring the complete dissolution of the deposited layer (Figure S6). Sine the generated Fe^3+^ can be readily reduced into Fe^2+^ again, the introduction of Fe^3+^/Fe^2+^ couple ensures a long-term effectiveness. As shown in Figure S7a and b, no any residual MnO_2_ deposits are detected on the working electrode after 150 cycles in this FeCl_3_-modified electrolyte, leaving the surfaces of the working electrode dense and uniform in both colored and bleached state (Figure S7c and d). As a result, the degradation of optical modulation amplitude is completely eliminated in this electrolyte (Figure 3b). In fact, the Δ*T*_550nm_ increases slightly from 44.8 % up to 46.9 % after 1000 cycles.

Besides increasing cycling stability, the addition of FeCl_3_ also improves the EC performance of this ROE-MnO_2_ system, thanks to the enhancement of reaction reversibility. As shown in Figure 3c–e, Δ*T*_550nm_ of this system increases from 39.4 % up to 45.0 % after FeCl_3_ addition, while the switching times and coloration efficiency keep comparable (Figure S8a and b). Table S2 systematically compares the performances of the RCI- and ROE-MnO_2_ EC systems, indicating that this ROE-MnO_2_ system delivers the best optical contrast.

**Figure 4:**
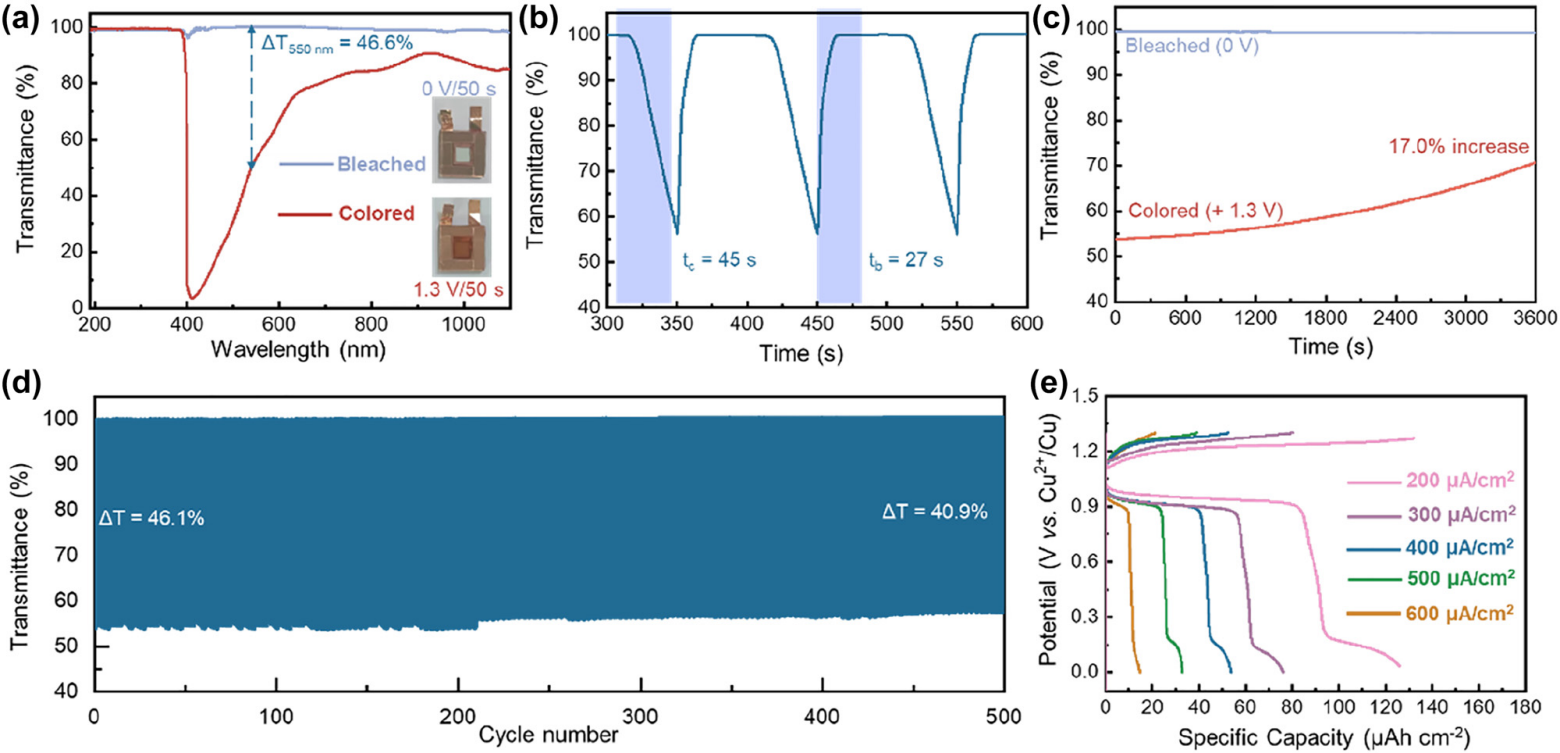
Electrochromic performance of the ROE-MnO_2_ dynamic window: (a) transmittance spectra and photographs (inset) in the colored and bleached states, (b) transmittance evolution curves showing the coloring/bleaching times, (c) transmittance evolution curves during open-circuit storage, (d) transmittance curves at 550 nm during continuous color switching. (e) GCD profiles at a verity of current densities.

To demonstrate the practical application of this ROE system, an EC device was assembled by using a FTO glass working electrode, a Cu foil counter electrode, and a FeCl_3_-modified electrolyte. Figure 4a shows the transmittance spectra and photographs (insets) of this device, which manifest an optical modulation amplitude of 46.6 % at 550 nm, and a coloration/bleaching time of 45/27 s, respectively (Figure 4b). Figure 4c further shown the optical memory ability of this device [50]. After 3600 s open-circuit storage, *T*_bleaching_ keeps unchanged, while *T*_coloring_ increased by 17.0 %, because of the current leakage caused by the reaction between Fe^2+^ and MnO_2_ (Figure S8c). As expected, the ROE-MnO_2_ dynamic window shows a decent lifetime with just a slight decrease of the optical modulation amplitude from 46.1 % to 40.9 % after 500 cycles (Figure 4d). Due to the largely different redox potentials of the working and counter electrode, this device shows also a noteworthy energy storage ability. As shown in Figure 4e, the device manifests a charge plateau of ca. 1.2 V and a discharge curve of ca. 1.0 V, in good line with the CV results (Figure 3a). The device exhibits a high capacity of 34.8 µAh cm^−2^ at a current density of 200 μA cm^−2^, 17 times higher than that of PB EC layer under the same current density [51]. As shown in Figure S9, two charged (i.e., colored) devices connected in series can successfully light up an LED, highlighting their promising application potential as electrochromic energy storage devices.

## 4 Conclusions

In summary, a novel electrochromic system based on reversible MnO_2_ electro-dissolution/deposition is established, in order to boost the optical performance of MnO_2_-based dynamic windows. This system achieves distinct bistable color change between rust brown and completely transparent, delivering much higher visible modulation amplitude than the traditional cation insertion/extraction MnO_2_ devices. By introducing Fe^3+^ into the electrolyte, cycling lifespan of this electrochromic system is effectively prolonged, thanks to the enhancement of MnO_2_ dissolution by the Fe^3+^/Fe^2+^ couple. We also assemble a reversible oxide electro-dissolution/deposition dynamic window, inorder to demonstratet the potential application of this electrochromic system.

## Supplementary Material

Supplementary Material Details
