# Supplementary material for: Visible light electrochromism based on reversible dissolution/deposition of MnO2
Source: Nanophotonics. 2023 Oct 30;13(5):679–86. doi: 10.1515/nanoph-2023-0573 (PMC11501562; doi:10.1515/nanoph-2023-0573)
Supplement: Supplementary file 1 — Supplementary Material Details [file j_nanoph-2023-0573_suppl_001.docx]

# Visible Light Electrochromism Based on Reversible Dissolution/deposition of MnO_2_

Xuan Liu^1,#^, Hanbing Wang^1,#^, Junsen Zhong^1^, Menghan Li^1^, Rui Zhang^1^, Dongjiang You^1^, Lingyu Du^1^,Yanfeng Gao^2,3,*^, Litao Kang^1,*^

^1^College of Environment and Materials Engineering, Yantai University, Yantai, 264005, China

^2^College of Materials Science and Engineering, Shanghai University, Shanghai, 200444, China

^3^Anhui Polytechnic University, Wuhu, Anhui 241000, China

^*^E-mail: yfgao@shu.edu.cn (Y.F. Gao); kanglitao@ytu.edu.cn (L.T. Kang);

^#^ X. Liu and H.B. Wang contributed equally to this paper and are co-first authors.

**Table S1.** Configurations and performances of typical RME devices.

| **Electrode** | | **Electrolyte** | **Tested cycles** | **Switching time (s)** | | **Voltage (V)** | **ΔT (%)@ Wavelength (nm)** | **Ref.** |
| --- | --- | --- | --- | --- | --- | --- | --- | --- |
| **Work** | **Counter** |  |  | **Bleach** | **Color** |  |  |  |
| Pt-ITO* | Cu | Cu(ClO_4_)_2_-LiClO_4_-HclO_4_-BiOClO_4_-H_2_O | 10,000 | 5 | 60 | -0.7/1.0 | 80@650 | [1] |
| Pt-ITO | Cu | AgClO_4_-Cu(ClO_4_)_2_-CuCl_2_-LiClO_4_-H_2_O | 5,000 | 70 | 180 | -0.6/0.8 | 76%@600 | [2] |
| Pt-ITO | Cu | CuCl_2_ -BiCl_3_-HCl-LiBr-PVA-H_2_O | 1,000 | 30 | 30 | -0.7/0.7 | 55%@550 | [3] |
| Pt-ITO | Cu | CuCl_2_-BiCl_3_- LiBr-HCl-HEC-H_2_O (gel) | 1,000 | 4 | 30 | -0.6/0.8 | 63%@600 | [4] |
| ITO | Ag | AgNO_3_-LiClO_4_-H_2_O | --- | 10.2 | 38.6 | -1.0/0.5 | 46@460 | [5] |
| ITO | ITO | AgNO_3_-LiBr-CuCl_2_-PVB-DMSO (gel) | --- | --- | --- | -4.0/-1.6 | --- | [6] |
| ITO | Zn | Zn(Ac)_2_-KCl-HEC-H_2_O (gel) | 2,500 | 30 | 40 | -1/1 | 78%@600 | [7] |
| Pt-ITO | Zn | ZnCl_2_-HCOONa-ZnBr-HEC-H_2_O (gel) | 250 | 90 | 30 | -0.8/2.3 | 60%@600 | [8] |

* Pt-ITO denotes Pt nanoparticle decorated ITO glasses.

**Table S2.** Comparison of the performance of ROE- and RCI-MnO_2_ EC systems.

| **System type** | **ΔT (%) @ Wavelength (nm)** | **Switching**  **time (s)** | | **Bias for switching (V)** | **Electrolytes** | **Tested cycles** | **Ref.** |
| --- | --- | --- | --- | --- | --- | --- | --- |
|  |  | **Bleach** | **Color** |  |  |  |  |
| RCI-MnO_2_ | 33.5@633 | 1.4 | 2.0 | --- | LiClO_4_-PC | 250 | [9] |
|  | 42.3@633  29.0@800 | 18.5 | 10.4 | -1.0/1.0 |  | --- | [10] |
|  | 9.0@680 | 12.3 | 8.1 | -0.4/0.4 *vs.* Ag/AgCl |  | 1,000 | [11] |
|  | 23.9@550 | 4.4 | | -2.5/3.0 |  | 1,000 | [12] |
|  | 30.0@410  3.3@680 | 7.9 | 6.8 | -1.8/1.5 | KCl-H_2_O | 500 | [13] |
| ROE-MnO_2_ | 89.7@420  45.0@550 | 24.0 | 45.0 | 1.3/0 | H_2_SO_4_-MnSO_4_-FeCl_3_-H_2_O | 1,000 | This work |


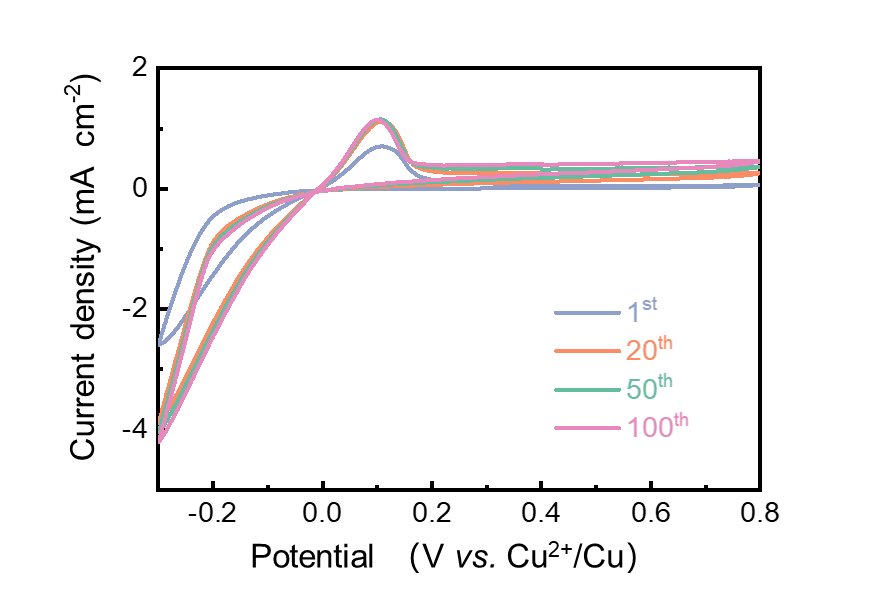


**Figure S1**. CV curves of a Cu/Ti asymmetric cell in 0.5 M H_2_SO_4_ + 15 mM CuSO_4_ electrolyte. The sweeping rate is 10 mV s^-1^.

In the CV tests, Ti, Cu foil, and 0.5 M H_2_SO_4_ + 15 mM CuSO_4_ aqueous solution were employed as the working, the counter electrode, and the electrolyte, respectively. As shown in **Figure S1**, the CV curves shows a pair of redox peaks, illustrating the successful plating/stripping of Cu in the electrolyte. In the first 20 cycles, the peak current density increased slightly, along with the narrowing of the polarization, indicating an improved Cu plating/stripping kinetics. Afterwards, the CV curves kept quite stable, suggesting a high cycling stability.


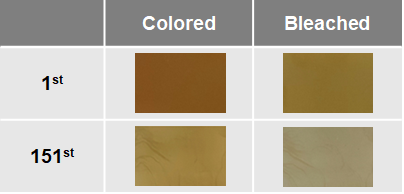


**Figure S2**. Optical photographs showing the colored and bleached states of the ROE-MnO_2_ system at the 1^st^ and 151^st^ cycles.


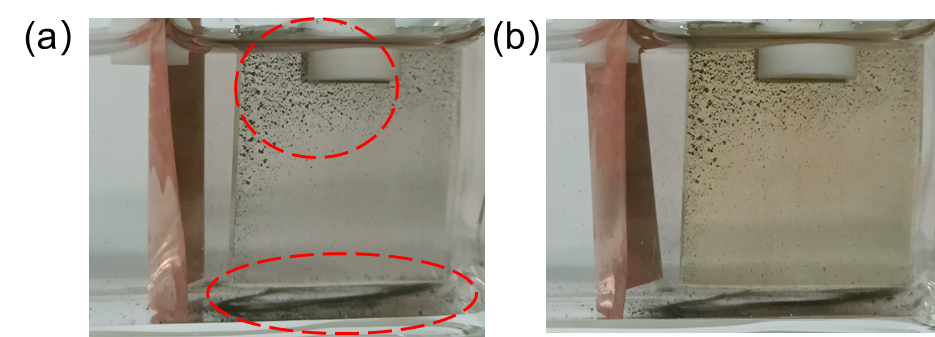


**Figure S****3**. Optical photographs of the ROE-MnO_2_ system in its (a) bleached and (b) colored state after 150 color switching cycles. After cycling test, numerous residual MnO_2_ deposits appear on the FTO glass substrate.


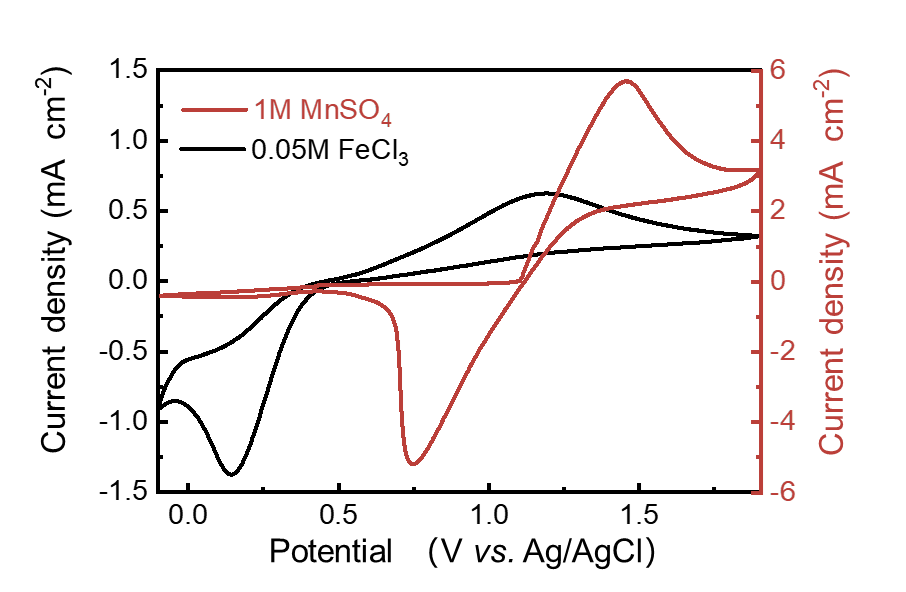


**Figure S4**. CV curves of an acidic MnSO_4_ and a FeCl_3_ electrolyte. The curves clearly show the lower redox potential of Fe^3+^/Fe^2+^ couple than the Mn^4+^/Mn^2+^ one.


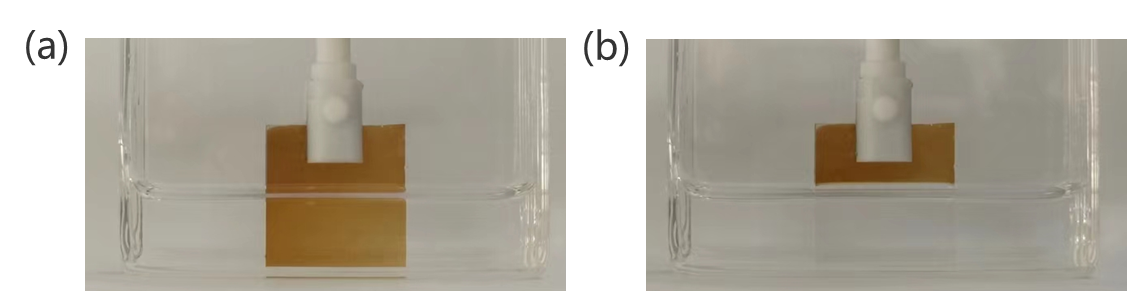


**Figure S5**. Optical photographs of the MnO_2_-coated FTO glass immersing in a 0.5 M H_2_SO_4_ + 15 mM FeSO_4_ solution: (a) initial state and (b) after 1 min.


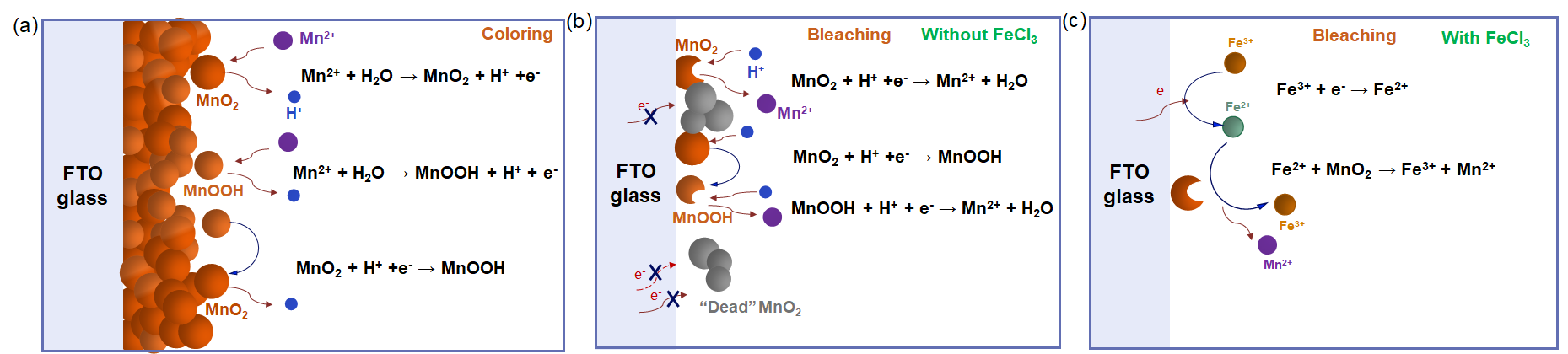


**Figure S6.** Schematical illustration showing the MnO_2_ deposition (a) and different dissolution behaviors in the unmodified (b) and 15 mM FeCl_3_ modified electrolyte (c). In panel (b), the “Dead” MnO_2_ refers to the residual isolated MnO_2_ that has lost electrochemical activity due to the electrically insulation.


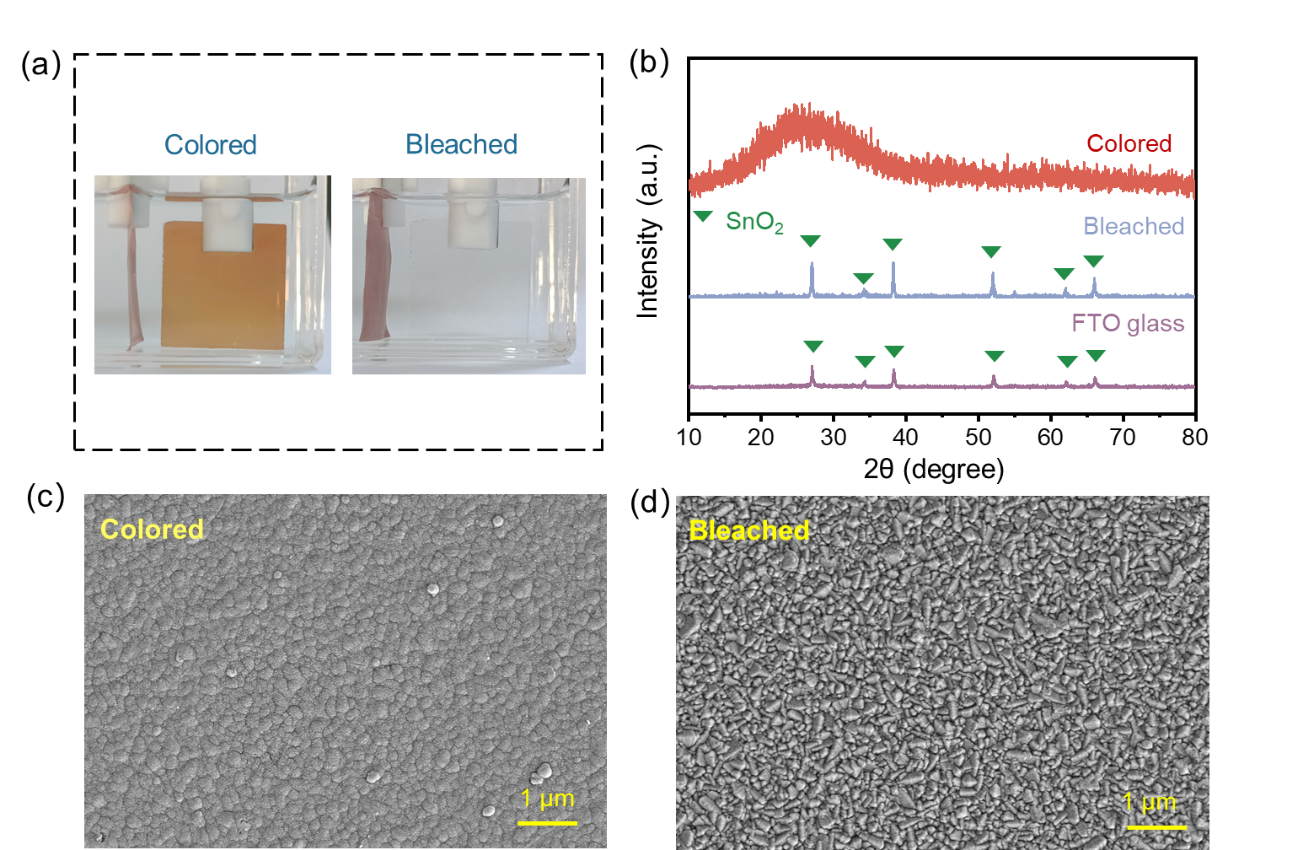


**Figure S7**. Cycling performance of the ROE-MnO_2_ system after 150 cycles in the FeCl_3_-modified electrolyte: (a) Optical photographs; (b) XRD patterns; (c-d) SEM images of the colored (c) and bleached (d) state.

As shown in Figure S7a, there are no any residual deposits on the working electrode after 150 cycles in the FeCl_3_-modified electrolyte, thanks to the enhancement of MnO_2_/MnOOH dissolution. XRD and SEM analyses also confirm the high reaction reversibility of this ROE-MnO_2_ system in the modified electrolyte. The surfaces of the FTO substrate and deposited layer maintain clean, dense and uniform after 150 deposition/dissolution cycles, indicating an impressive cycling durability.


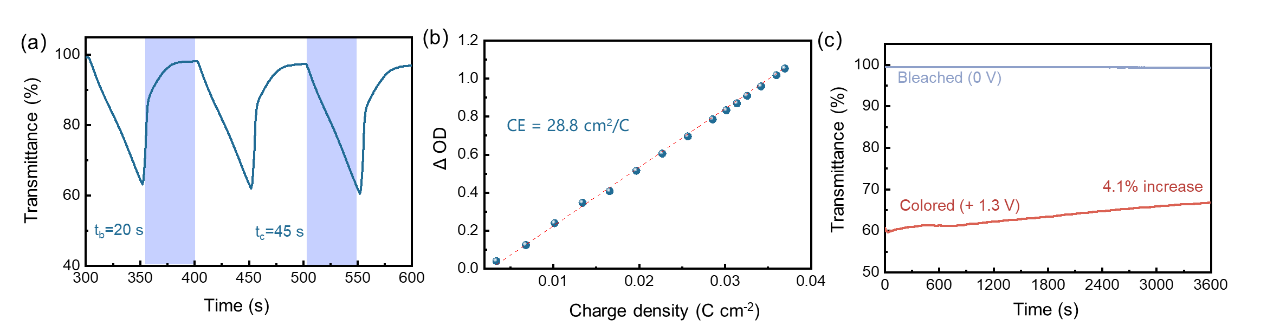


**Figure S8**. Electrochromic performance of the ROE-MnO_2_ system in the unmodified electrolyte: (a) Transmittance evolution curve showing the coloring/bleaching times. (b) optical density (ΔOD, @550 nm) versus charge density curves, (c) transmittance evolution curves during open-circuit storage.


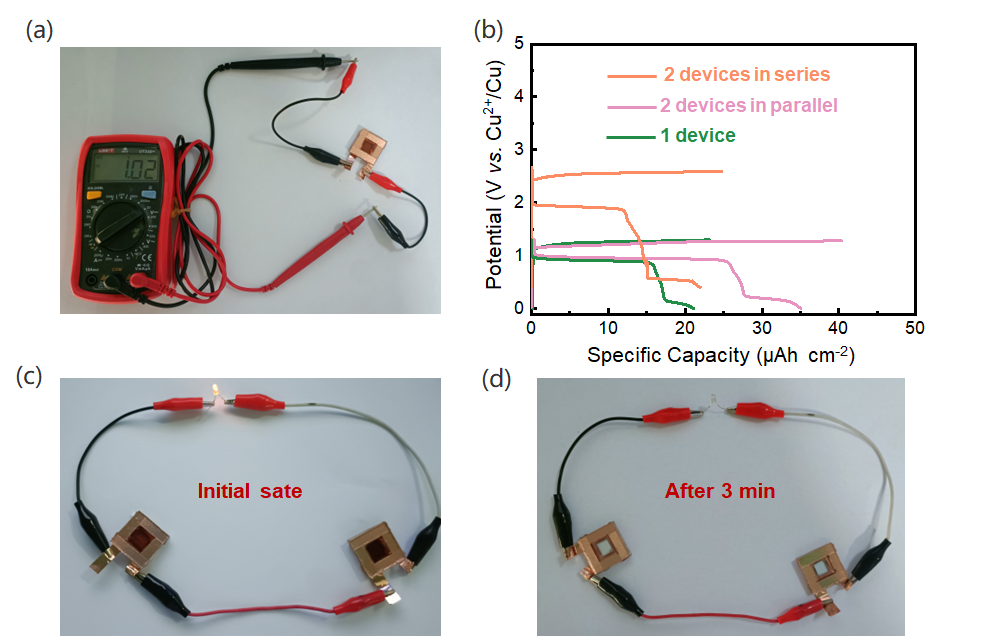


**Figure S9**. (a) The open-circuit voltage of a fully charged ROE-MnO_2_ EC device, (b) electrochromic performance of the devices connected in different ways, (c-d) an LED light powered by two devices connected in series.

As shown in **Figure S9a**, a single device outputs an open-circuit voltage of 1.02 V. To mimic the practical application conditions, the electrochromic performance of the devices connected in different ways are also tested. At all conditions, the devices deliver expected capacity and voltage outputs (**Figure S9b**). Therefore, two charged (i.e., colored) devices connected in series can successfully light up an LED (**Figure S9c**). After 3 min, the devices transform into full transparent due to the depletion of charge (**Figure S9d**), highlighting the promising application potential of the ROE-MnO_2_ systems as electrochromic energy storage devices.

## References

1. Hernandez, T.S., et al., *Electrolyte for Improved Durability of Dynamic Windows Based on Reversible Metal Electrodeposition.* Joule, 2020. **4**(7): p. 1501-1513.

2. Barile, C.J., et al., *Dynamic Windows with Neutral Color, High Contrast, and Excellent Durability Using Reversible Metal Electrodeposition.* Joule, 2017. **1**(1): p. 133-145.

3. Strand, M.T., et al., *Polymer inhibitors enable >900 cm2 dynamic windows based on reversible metal electrodeposition with high solar modulation.* Nature Energy, 2021. **6**(5): p. 546-554.

4. Hernandez, T.S., et al., *Bistable Black Electrochromic Windows Based on the Reversible Metal Electrodeposition of Bi and Cu.* ACS Energy Letters, 2017. **3**(1): p. 104-111.

5. Zhang, W., H. Li, and A.Y. Elezzabi, *Nanoscale Manipulating Silver Adatoms for Aqueous Plasmonic Electrochromic Devices.* Advanced Materials Interfaces, 2022. **9**(19).

6. Onodera, R., et al., *Coloration mechanisms of Ag deposition-based multicolor electrochromic device investigated by morphology of Ag deposit and its optical properties.* Journal of the Society for Information Display, 2016. **24**(7): p. 424-432.

7. Islam, S.M. and C.J. Barile, *Dynamic Windows Using Reversible Zinc Electrodeposition in Neutral Electrolytes with High Opacity and Excellent Resting Stability.* Advanced Energy Materials, 2021. **11**(22).

8. Madu, D.C., et al., *Electrolytes for reversible zinc electrodeposition for dynamic windows.* Journal of Materials Chemistry C, 2021. **9**(19): p. 6297-6307.

9. Su, Y., et al., *A dual-function device with high coloring efficiency based on a highly stable electrochromic nanocomposite material.* Chemical Engineering Journal, 2023. **456**.

10. Zhao, L., et al., *High-performance complementary electrochromic energy storage device based on tungsten trioxide and manganese dioxide films.* Sustainable Materials and Technologies, 2022. **32**.

11. Zhou, D., B. Che, and X. Lu, *Rapid one-pot electrodeposition of polyaniline/manganese dioxide hybrids: a facile approach to stable high-performance anodic electrochromic materials.* Journal of Materials Chemistry C, 2017. **5**(7): p. 1758-1766.

12. Sajitha, S., U. Aparna, and B. Deb, *Ultra‐Thin Manganese Dioxide‐Encrusted Vanadium Pentoxide Nanowire Mats for Electrochromic Energy Storage Applications.* Advanced Materials Interfaces, 2019. **6**(21).

13. Ma, D., et al., *Wide-Spectrum Modulated Electrochromic Smart Windows Based on MnO(2)/PB Films.* ACS Appl Mater Interfaces, 2022. **14**(1): p. 1443-1451.
